# The feasibility of the COMMUNICATE toolkit to support the communication of physical activity messages with adolescents in Irish schools

**DOI:** 10.1093/heapro/daag110

**Published:** 2026-08-06

**Authors:** Caera L Grady, Elaine Murtagh, Kwok Ng, Enrique García Bengoechea, Catherine B Woods

**Affiliations:** School of Sport and Human Performance, Faculty of Science and Health, Dublin City University, Dublin, Ireland; Physical Activity for Health Research Centre, Health Research Institute, University of Limerick, Limerick, Ireland; Department of Physical Education and Sport Sciences, Faculty of Education and Health Sciences, University of Limerick, Limerick, Ireland; Physical Activity for Health Research Centre, Health Research Institute, University of Limerick, Limerick, Ireland; Department of Physical Education and Sport Sciences, Faculty of Education and Health Sciences, University of Limerick, Limerick, Ireland; Department of Teacher Education, Turku Institute for Advanced Studies, University of Turku, Turku, Finland; Institute of Sports Science and Innovation, Lithuanian Sport University, Kaunas, Lithuania; Physical Activity for Health Research Centre, Health Research Institute, University of Limerick, Limerick, Ireland; Department of Physical Education and Sport Sciences, Faculty of Education and Health Sciences, University of Limerick, Limerick, Ireland; Physical Activity for Health Research Centre, Health Research Institute, University of Limerick, Limerick, Ireland; Department of Physical Education and Sport Sciences, Faculty of Education and Health Sciences, University of Limerick, Limerick, Ireland

**Keywords:** acceptability, appropriateness, adolescents, messages, health literacy

## Abstract

The Active School Flag (ASF) is a peer-led whole-of-school physical activity (PA) programme in Irish secondary schools. This study assessed the feasibility of implementing the COMMUNICATE toolkit for ASF programme implementers (peer leaders and coordinating teachers). A pre-post, quasi-experimental, mixed-methods design was employed and guided by the Orsmond and Cohn feasibility objectives. Online surveys, semi-structured interviews, and focus groups were collected from six secondary schools (40% recruitment rate), 170 peer leaders, and three coordinating teachers. Survey data were analysed using descriptive statistics and independent and paired samples *t*-tests. Interview and focus group data were analysed using a combined inductive–deductive reflexive thematic analysis. Data were integrated during the writing-up stage. The toolkit was acceptable and appropriate; however, full implementation was hindered by time constraints within the busy school schedule and short implementation period. Methodological refinements regarding data collection timing and instrument validity and reliability were identified as necessary. Despite no differences between the intervention and control groups’ communication skills or health knowledge at follow-up, the toolkit positively impacted peer leaders’ personal development and knowledge and the PA and knowledge levels of their peers. While a real-world implementation design, led by programme implementers, may improve intervention sustainability and cost efficiency, implementation support and flexibility in delivery are essential. The COMMUNICATE toolkit was a valuable resource for whole-of-school programme implementers. Nevertheless, changes to the intervention and study procedures are required before proceeding to a definitive trial. Future research should consider an internal pilot within a definitive trial to ensure the changes are sufficient.

Contribution to Health Promotion

- Provides clear evidence on the feasibility of integrating a co-created communications toolkit into an existing whole-of-school programme, ultimately informing future definitive trial designs.
- Outlines specific refinements to data collection procedures and study design when evaluating complex, school-based interventions.
- Offers insight into the real-world practicalities of school-level implementation regarding barriers and facilitators while highlighting the necessity to consider context and offer intervention flexibility.
- Emphasizes the importance of iteratively tailoring health promotion interventions with the relevant actors (e.g. teachers, students, school management) to ensure sustainability and relevance within each school as its own subsystem of the education system.

## Introduction

Physical inactivity among adolescents, comprising 16% of the global population, remains a critical public health challenge (Guthold *et al.* 2020, UNICEF 2022). Adolescence is a prime opportunity to intervene as physical activity (PA) habits develop and track into adulthood (Hayes *et al.* 2019). The majority of children and adolescents’ waking hours revolve around the school setting. Consequently, schools are one of the societal institutions with the largest reach and influence over a population group for an extended period of their lifespan, making them ideal for PA promotion (Kriemler *et al.* 2011). However, schools are not without their challenges. The busy, complex and constantly evolving nature of schools within the wider education system makes implementing school-based interventions challenging (Daly-Smith *et al.* 2020). Factors like timing, policy gaps, and poor communication often hinder success (McHale *et al.* 2022). Researchers have called for an asset-based, multi-component whole-of-school approach to school-based PA promotion (Milton *et al.* 2021, García Bengoechea *et al.* 2024).

A whole-of-school programme (WSP) is a multi-component approach with a commitment to promoting PA to all members of the school community through enabling school policies, environment and sustainable opportunities (Milton *et al.* 2021). Examples of international WSP’s include Finnish Schools on the Move (Finland), Creating Active Schools (UK), Let’s Move Active Schools (US), and Active School Flag (ASF; Ireland; Daly-Smith *et al.* 2020, McMullen *et al*. 2022).

In Ireland, the second-level ASF is a multi-stage peer-led PA-WSP with student voice and peer leadership at its core (Ng *et al.* 2019). It has three stages: an introduction where schools fulfil the minimal criteria, a certificate where schools focus on improving in school PA opportunities, and a flag where schools focus on promoting community-based PA opportunities. The implementers include a class of peer leaders (typically 15–16 years) and coordinating teachers. The ASF class is embedded into the school timetable, typically for 40–60 min, once or twice per week. The peer leaders’ roles and responsibilities include conducting a whole-of-school PA audit and developing an action plan to create more PA opportunities. In addition, peer leaders deliver a ‘Did You Know?’ communications campaign to raise awareness about the benefits of and opportunities for PA (Ng *et al.* 2019, Grady *et al.* 2025b). This ‘Did You Know?’ campaign involves developing a communications strategy to keep the school community up to date with campaigns, initiatives, and events. All ASF schools are provided with standard guidelines outlining the purpose of the campaigns, suggested messages, important factors to consider (e.g. the mode of communication), evaluating the impact, and some steps to creating an informative campaign (Supplementary File 1). However, a recent examination of the ‘Did You Know?’ communications campaign indicated that the guidelines were insufficient for implementers to successfully deliver a campaign and to create a meaningful change (Grady *et al.* 2025b).

Communication campaigns are one of the key actions recommended in the Global Action Plan for PA to raise awareness of the health benefits of PA and promote behaviour change by 2030 (World Health Organisation 2018). A PA message can be defined as ‘educational or persuasive material’ that is communicated to people with the aim of improving PA levels (Williamson *et al.* 2020). There is a need to standardize the methods used to communicate and evaluate PA messages involving young people and embedding communication campaigns within existing WSPs can maximize their impact (Grady *et al.* 2025a). The COMMUNICATE toolkit was co-created with, and for use by, WSP implementers, especially peer leaders, to support their efforts to communicate PA messages within the school. The toolkit targets multiple levels and adopts systems thinking. The toolkit highlights the importance of raising awareness about PA and the WSP, planning and maximizing communication efforts, and provides the necessary training for developing peer leaders’ communication skills (Grady *et al.* 2025c). The COMMUNICATE toolkit is detailed in the methods section.

Following intervention development, feasibility testing is the next step in complex intervention development to determine any further necessary adaptations to the intervention before proceeding to a definitive trial (Skivington *et al.* 2021). The interchangeable use of the terms feasibility and pilot has raised issues for researchers and reviewers (Orsmond and Cohn 2015). Feasibility studies have a flexible methodology and are iterative, formative, and adaptive (Dobkin 2009). Orsmond and Cohn (2015) outline five objectives for feasibility studies: the evaluation of the recruitment process and sample characteristics; data collection procedures and outcome measures; intervention and study procedure acceptability and suitability; resources, management, and implementation of the study and intervention; and evidence of promise for the intervention. This study utilized the Orsmond and Cohn (2015) objectives to assess the feasibility of the COMMUNICATE toolkit in supporting WSP implementers with PA communication campaign delivery.

## Materials and methods

The Consolidated Standards of Reporting Trials (CONSORT) 2010 statement extension for pilot or feasibility studies was followed (Supplementary File 2; Eldridge *et al.* 2016). A pre-post, quasi-experimental, sequential mixed-methods study design was adopted. Data were integrated during analysis and write-up. Ethical approval was obtained from the University of Limerick Education and Health Sciences Research Ethics Committee before the study commenced (2018_10_18_EHS).

### Participants

The ASF Stage 2 schools were identified as the target group for this study as they had at least one year of experience in delivering the programme and may benefit the most from additional resources. As the overall ASF programme was undergoing development and pilot testing at the time of this study, there were only 15 schools in total in Stage 2 across Ireland. Purposive sampling was used to recruit at the school level through the ASF programme. A recruitment email was circulated to all Stage 2 ASF schools (*N* = 15) inviting the ASF peer leaders and coordinating teachers to participate in a study assessing the feasibility of the COMMUNICATE toolkit to support the implementation of the ASF communications campaign. Of the schools invited, 11 were mixed gender and 4 were single-gender girls’ schools. Schools were eligible if they were enrolled in the second stage of the ASF programme, had a weekly timetabled ASF class for peer leaders, and were able to engage within the timeframe, committed to completing study measures and the ASF coordinating teacher agreed to deliver the intervention.

**Figure 1:**
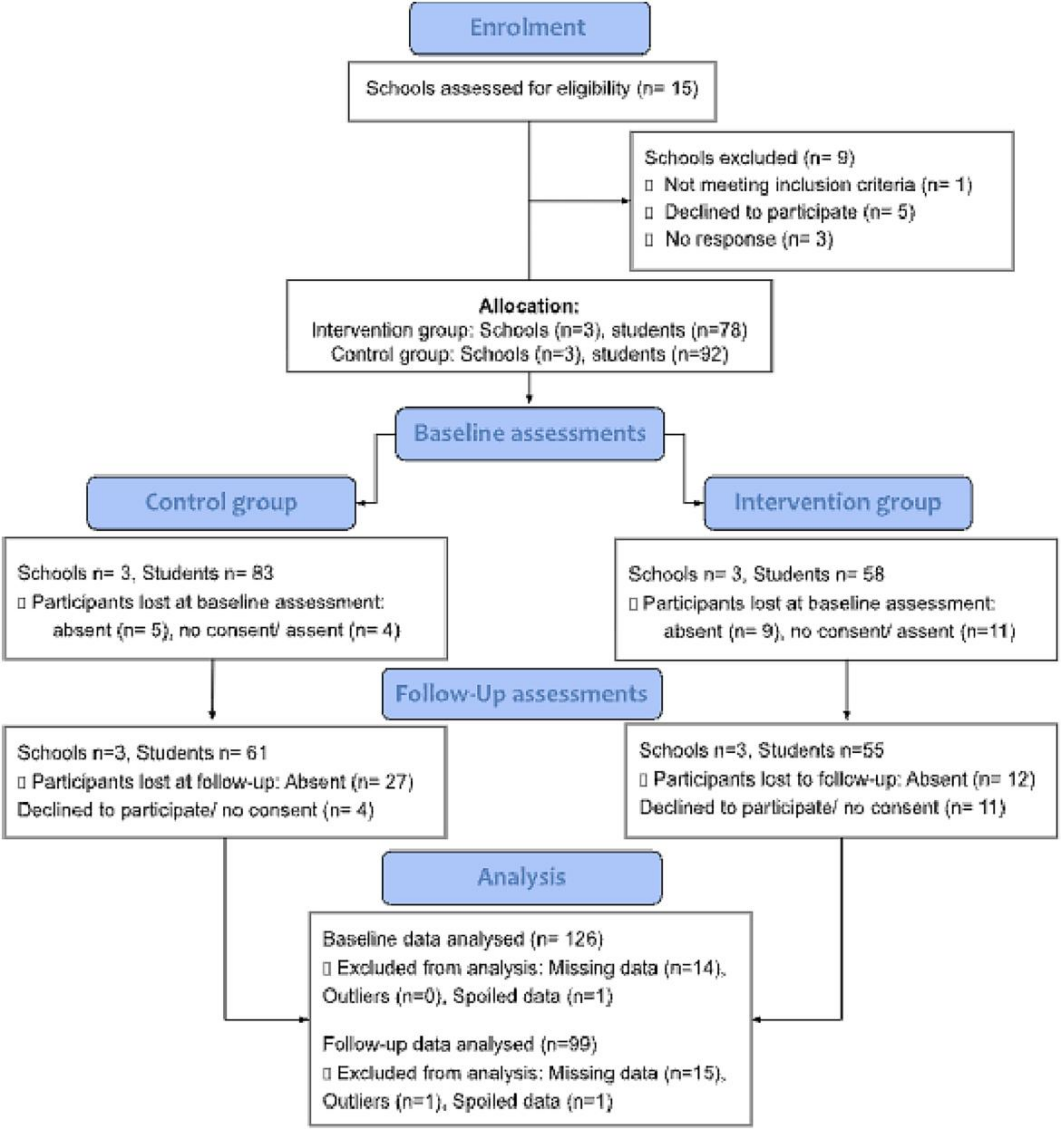
Participant flow diagram.

Of the schools that responded (*N* = 12), six were eligible and willing to participate in the study (Fig. 1). Once eligibility at the school level was confirmed, the ASF programme implementers, including the class of peer leaders and the coordinator, were invited to participate in surveys, semi-structured interviews, or focus groups. The ASF class of peer leaders were in Transition Year (fourth year, typically 15–16 years old), a non-academic gap year between junior and senior cycle in Ireland where students get an opportunity to learn and gain experience outside of the traditional subjects (Clerkin 2012). Recruitment lasted 4 weeks between October and November 2024. Three schools were assigned to the intervention group based on proximity to the university and ability to engage with study requirements. The remaining three schools were assigned to the control group. A total of 170 ASF peer leaders across the six schools were invited to participate. Three ASF coordinators from the intervention schools participated.

### Intervention group

In addition to the standard ASF guidance for the communications campaign, the intervention group coordinating teachers received the COMMUNICATE toolkit to support the peer leaders in communicating PA messages. The COMMUNICATE toolkit was co-created with students and teachers from ASF schools during the 2023–2024 academic year (Grady *et al.* 2025c). The toolkit was tailored to the ASF programme to ultimately complement its implementation. The toolkit includes a teacher’s manual, a student slideshow, and a box of resources and tools for the ASF coordinator to use with the peer leaders. The COMMUNICATE toolkit aims to increase the peer leaders’ skills and competencies for communicating PA messages through raising awareness about PA and ASF, increasing capacity, and recognizing best practices for communicating PA messages. The toolkit contains 19 different activities to be completed throughout the year (Grady *et al.* 2025c). The COMMUNICATE toolkit is described in full elsewhere; see Grady *et al*. (2025c) for a full description and overview of the COMMUNICATE Toolkit.

Before schools were allocated to the intervention arm, the ASF coordinator confirmed, via email, their commitment to attend an introduction session before the baseline assessments, monthly check-in meetings, and a final debrief interview about the intervention. The ASF coordinating teacher was asked to use as many elements of the toolkit as possible to support ASF implementation throughout the year. Monthly check-ins were established to understand the implementation of the COMMUNICATE toolkit and avoid recall bias at the end of the intervention. A fidelity checklist guided the monthly check-ins (Supplementary File 3). The Orsmond and Cohn (2015) feasibility objectives (Supplementary File 4) guided the semi-structured interview script.

### Control group

The control group was also in the ASF programme. Thus, they were required to run a communications campaign and received the basic guidance for running these campaigns (Supplementary File 1). The control group did not receive the additional support for running the communications campaign. Peer leaders from the control group participated in the pre- and post-intervention surveys.

### Procedure

All participants, parents, or guardians received a participant information sheet before participating in this study. Participants were informed that this study was voluntary and that they could change their mind about participation at any time up until pseudonymization took place at the end of the study. Passive, informed parental or over-16 consent was used for the low-risk surveys (i.e. if you wish to participate, no further action is required), and active, informed parental or over-16 consent was gained (i.e. written and signed agreement to participate) for the focus groups and interviews. Peer leaders informed assent was gained at the beginning of all assessments.

Assessments occurred at baseline and follow-up for both groups. All surveys were hosted on the Qualtrics survey platform. Each coordinating teacher received a personalized link to share the survey with the peer leaders. Survey responses were linked between baseline and follow-up using a unique student ID code. The ASF coordinating teacher administered the student surveys during the timetabled class in November 2024 and May 2025. Intervention schools received additional questions about the COMMUNICATE toolkit at follow-up. The intervention group ASF coordinating teachers completed an anonymous survey after the intervention, assessing the feasibility, acceptability, and appropriateness of the COMMUNICATE toolkit (June 2025).

At follow-up, a subsample of the intervention group was invited to participate in a focus group or interview with the first author (C.L.G.). The ASF coordinating teachers in each school asked peer leaders to volunteer to participate in a focus group after the final survey with members of the research team. The research team explained the purpose and emphasized that participation was voluntary and there were no consequences if they chose not to participate.

**Figure 2:**
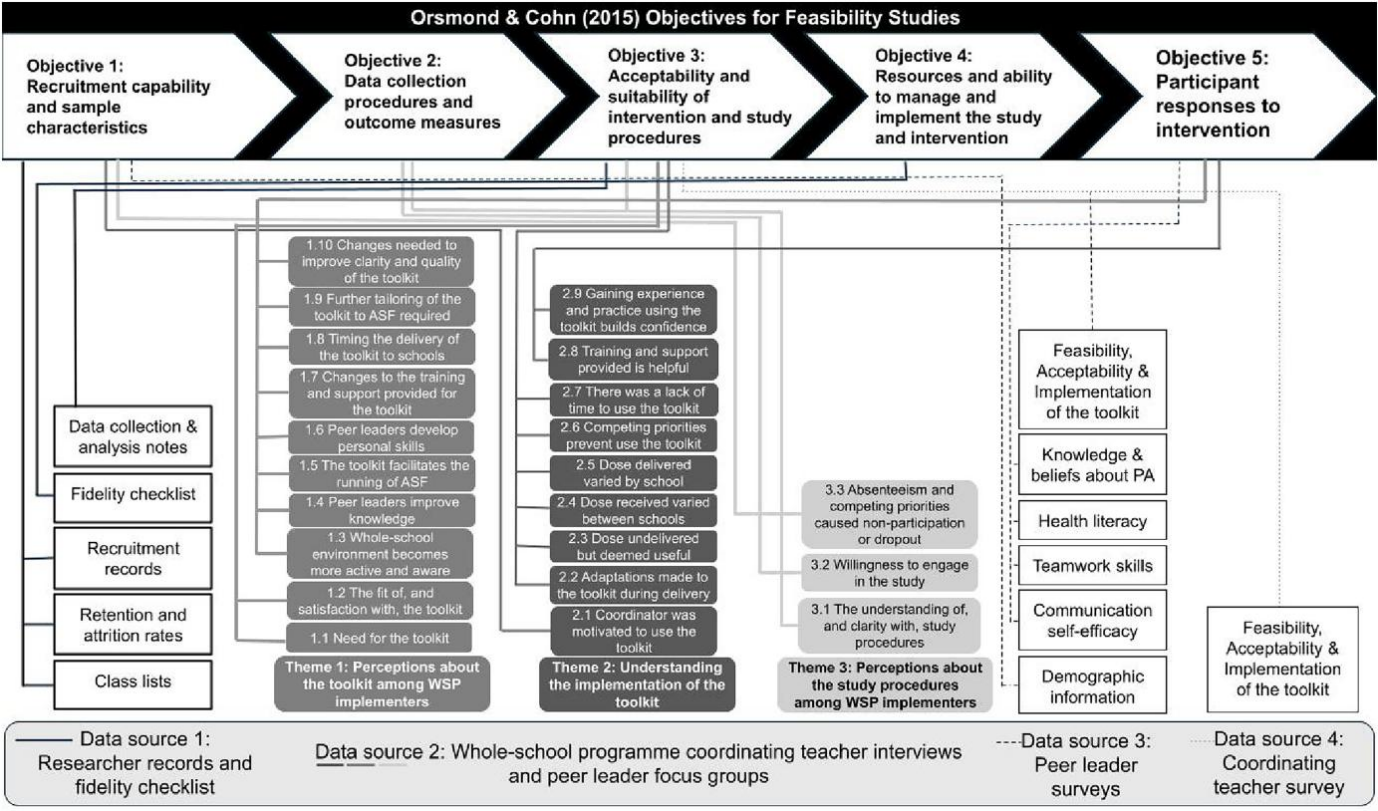
Graphic thematic map of the interview data.

The interviews and focus groups were conducted by the first author (C.L.G.) who has a wealth of experience with qualitative research methods (McHale *et al.* 2022, Murphy *et al.* 2023, Grady *et al.* 2025b, 2025c). An assistant moderator (DC) was present for the focus group discussions to take notes, summarize the discussion, and ask any additional questions. All focus groups and interviews were recorded and transcribed using the MS Teams record and transcribe function and were independently checked and cleaned by the first author. Student focus groups took place in person within each respective school, during normal school hours. Coordinating teachers were also invited to participate in an interview online or in-person and during or outside school hours. Once analysis was completed, prior to the writing-up stage, all data were integrated by mapping each data point appropriately to the corresponding feasibility objective. Figure 2 visually presents the mapping process.

### Feasibility outcomes

To assess the feasibility of the COMMUNICATE toolkit, data sources were predefined (Supplementary File 5). These included researchers’ records of recruitment and retention of schools and participants and a fidelity checklist (completed by the researcher during monthly check-ins), post-intervention interviews with coordinating teachers and focus groups with peer leaders, pre-post intervention surveys with peer leaders, and a post-intervention survey with coordinating teachers.

The Weiner *et al.* (2017) implementation questionnaire assessed the feasibility, acceptability, and appropriateness of the COMMUNICATE toolkit. Three four-item subscales on a 5-point Likert scale (1 ‘completely disagree’ to 5 ‘completely agree’) measured feasibility, acceptability, and appropriateness. The questionnaire has not yet been validated with the adolescent population but has a reading age of fifth grade, typically 10–11 years. A mean score was calculated for each scale.

### Instruments to assess intervention outcomes

The survey collected demographic information, including gender, grade, and socio-economic status. Participants were asked to report if they identified as male, female, or another gender. School type was collected from the Department of Education’s school list details from their website (Department of Education and Skills 2025). The Family Affluence Scale III—developed for, and psychometrically validated with, school-aged children—assessed the socio-economic status of the peer leaders (Torsheim *et al.* 2016).

Peer leaders’ self-efficacy to communicate PA was assessed using an 8-item communication self-efficacy measure, on a 10-item Likert scale (1 ‘cannot do at all’ to 10 ‘highly certain can do’) adapted from a survey for health professionals communicating with individuals with disabilities (Lassiter *et al.* 2023). Teamwork skills were assessed using a student self-report teamwork 30-item Likert scale (1 ‘never’ to 6 ‘always’; Zhuang *et al.* 2008). The 10-item health literacy scale for school-aged children assessed the peer leaders’ knowledge and competencies to promote health (Paakkari *et al.* 2016). Sum scores were calculated for each of the self-efficacy, teamwork, and health literacy scales. Knowledge of PA and beliefs about the consequences of behaviour were assessed using two subscales from the Determinants of PA Questionnaire on a 7-point Likert scale (1 ‘strongly disagree’ to 7 ‘strongly agree’) (Taylor *et al*. 2013). A mean score was generated for these subscales.

### Data analysis

Survey data were entered and analysed in the Statistical Analysis Software Package SPSS (version 29.0.2.0). Participants with large amounts of missing data and outliers were removed before analysis. If items within a given scale were left unanswered, these were treated as missing data, and a scale sum score was not computed for the respective participant. Consequently, their data for that specific scale were excluded from further analysis. Descriptive statistics were run for participants’ characteristics and the outcome measures using percentages, means, and standard deviations. The data were analysed, comparing both within and between-group means. A paired samples *t*-test was conducted to establish the differences in means from baseline to follow-up, and the effect size was calculated using Cohen's *d*. An independent sample *t*-test was used to determine differences between group means at baseline and follow-up, and effect sizes were calculated using Hedges’ *g*.

Interview and focus group data were transcribed verbatim and analysed using a combined inductive–deductive reflexive thematic analysis (Braun and Clarke 2019). Reflexive thematic analysis allows organic and open procedures for coding and theme development that relies on the researcher’s interpretative engagement with the data (Braun and Clarke 2024). The first author positions herself as a critical realist and a pragmatist, thus allowing the research question to guide the selection of data collection and analytical methods. The first author practised reflection by questioning herself and reflexivity by questioning others and the data. Noting thoughts, feelings, interpretations and assumptions and discussing these with co-authors from conceptualization to write-up facilitated this reflexive process. Data were pseudonymized before being entered and analysed in NVivo (version 1.7.2). The first author was involved at all stages of the conceptualization, collection, and analysis process, which facilitated familiarization with the data. An iterative process of open coding was followed, whereby the data in each transcript were given semantic codes until all transcripts were fully coded. An initial process of grouping codes was conducted before mapping the data to the Orsmond and Cohn (2015) framework. This process involved critically assessing how each data point fit with the appropriate objective that best described the data. The resulting mapped data were discussed with the remaining authors to ensure a fit between the data point and the corresponding feasibility objective.

## Results

### Feasibility outcomes

The following section outlines and integrates the findings from the researchers’ records, survey, and interview data. Figure 2 presents a graphic map of the various data sources demonstrating the spread of the participants’ responses across the five objectives. The findings are presented under the five feasibility objectives, integrating the data from the four different sources, including the 3 themes and 22 subthemes generated from the interview and focus group data.

### Objective 1: evaluation of recruitment capability and resulting sample characteristics

Out of 15 eligible schools, six progressed to the trial (40% recruitment rate). Reasons for not participating or ineligibility are outlined in Fig. 1. The one ineligible school did not have a timetabled ASF class of peer leaders. Of the six participating schools, three were single-gender girls and three were mixed gender. Mixed-gender schools accounted for a 73% majority in the eligible sample. Table 1 outlines the school characteristics and the recruitment and retention rates of the peer leaders. There were no single-gender boys’ schools within the sampling pool. One was a socio-economically disadvantaged status school of the six in the total eligible sample.

**Table 1:** School characteristics and peer leader recruitment and retention rates.

Peer leader recruitment and retention rates									
School information					Baseline		Follow-up		
School #	School type	Intervention or control group	Total no. of peer leaders in class	Total *n* with no consent/assent	Total *n* absent	Survey response rate (*n*)^a^	Total *n* absent	Survey response rate (*n*)	Focus group participation (*n*)
1	Mixed, rural	Intervention	28	3	4	20	2	22	9
2	Single gender: girls urban, DEIS	Intervention	23	5	0	18	6	14	9
3	Single gender: girls, Rural	Intervention	27	2	5	20	4	19	0
Intervention group totals			78	11	9	58	12	55	18
4	Single gender: girls, rural	Control	26	1	0	23	4	17	N/A
5	Mixed, rural	Control	28	3	5	22	10	18	N/A
6	Mixed, rural	Control	38	0	0	38	6	26	N/A
Control group totals			92	4	5	83	27	61	N/A
Total (intervention and control group)			170	15	14	141	39	116	18
Percentage of total peer leaders (*N* = 170)				8.8	8.2	82.9	22.9	68.2	10.6

^a^Number of responses after initial cleaning (i.e. removing duplicates, etc.) and before data analysis

The survey was completed by 83% of eligible peer leaders at baseline (*N* = 141), and there was an 82% retention rate at follow-up. Reasons for drop off at follow-up included a ‘hectic’ schedule and absence on the day of assessment. Before analysis several cases were removed due to missing data, spoilt data, or outliers (Fig. 1). Participants reported survey fatigue due to the prevalence of school-based research identifying it as a primary barrier to completion.

Eighteen peer leaders from two intervention schools participated in focus groups at the follow-up assessment. The remaining school was unable to participate in the focus groups due to unforeseen circumstances (i.e. coordinating teacher on sick leave). The three coordinating teachers in the intervention schools completed all follow-up assessments.

The average age of peer leaders at baseline was 15.6 ± 0.73 years; 57.9% identified as female; and 69% had a moderate family affluence rating. At follow-up, the average age was 15.9 ± 0.42 years, the majority identified as female (66.7%), and 58.2% had a moderate family affluence rating. See objective five for further descriptive statistics of the sample.

Post-intervention, one control school (School 6) disclosed that they operated a rotating class of peer leaders whereby all students in the year group had an opportunity to be a peer leader. Therefore, those surveyed pre-intervention were only peer leaders for the first half of the academic year. To allow for within-group comparison, the same peer leaders were surveyed at follow-up despite only being exposed to half of the ASF programme. A rotating ASF class was not established as an eligibility criterion before study commencement and should be added for the future.

### Objective 2: evaluation and refinement of data collection procedures and outcome measures

Peer leader took on average 20.95 ± 9.4 min at baseline and 21.58 ± 9.36 min at follow-up to complete the survey. Interviews and focus groups took on average 40 min. The coordinating teachers felt that the time commitment involved with data collection was manageable, but flexibility is essential due to the busy school environment and other commitments.

It wasn’t a huge [amount of time involved] for me, quite a bit of time with the [teams] calls (the coordinating teacher interviews) but that’s the nature of it, but the surveys, I think can be done in your own time. (Coordinating teacher B, male, School 2)

Both peer leaders and the coordinating teachers understood the importance and relevance of surveys ‘to develop the programme’ (Coordinating teacher C, male, School 3) and ‘it is the easiest way because you can get feedback often and you can put whatever you want to know in it’ (Student 5a, male, School 1). Some peer leaders felt that ‘the time involved [to fill out the survey] is a lot’ (Student 6b, female, School 2), and there was some confusion amongst the peer leaders in relation to which survey was being referred to, further evidencing the presence of survey fatigue. In relation to the clarity of the procedures, coordinating teachers were ‘able to understand what was going on and what you were looking for’ (Coordinating teacher B, male, School 2); however, they felt that the purpose of doing the surveys ‘often goes over their heads’ (Coordinating teacher A, female, School 1). There were no specific concerns expressed about the main outcome measures of the study.

All scales, except for one, had a Cronbach’s *α* > 0.7, indicating acceptable or good internal consistency (Supplementary File 6). The Determinants of PA Questionnaire knowledge of PA scale had poor internal consistency (T1 *α* = 0.426; T2 *α* = 0.593). Upon removal of the only positively worded item in the scale, the score was closer to an acceptable range (T1 *α* = 0.563, T2 *α* = 0.740). The magnitude and practical significance of the data were assessed using a paired *t*-test, and effect sizes were calculated using Cohen's d statistic. All items increased from baseline to follow-up in both groups; however, only the communication self-efficacy scale had a large effect size (*P* < .001, *d* = 1.66); the remaining had small effect sizes (*d* < 0.02). There were no major reported issues with these instruments, although some concerns were expressed, ‘there were a lot of questions…or some random questions in that regard that they really didn’t understand any of that…the specific questions about their home life [Family Affluence Scale] and [the peer leaders] were like, “but this is an active school survey”’ (Coordinating teacher C, male, School 3).

### Objective 3: evaluation of acceptability and suitability of intervention and study procedures

All three coordinating teachers agreed that the toolkit fits well within the ASF programme and the Transition Year programme.

The new [Transition Year, i.e. fourth year in Irish secondary schools, academic gap year between junior and senior examination cycles] programme coming in now kind of talks a lot about reflection, and you know, there is an awful lot of learning for [Transition Year] students in the active school programme. Forget about the idea of physical activity, but just the basic skills that they get out of it in terms of communication, organisation, all that sort of thing. So it does, it links in a lot with the principles of [Transition Year]. (Coordinating teacher C, male, School 3)

The coordinating teachers were motivated to use the toolkit as it was an extra resource ‘like basically, having a book to refer to’ (Coordinating teacher B, male, School 2) that provided practical activities and facilitated the coordinating teachers’ role in the ASF class.

The scores from the acceptability, appropriateness, and feasibility of the COMMUNICATE toolkit questionnaire from the peer leaders (*n* = 56) and the coordinating teachers (*n* = 3) are presented in Table 2.

**Table 2:** Acceptability, appropriateness, and feasibility of the COMMUNICATE toolkit.

	Overall (*N* = 59)		Student (*N* = 56)		Teacher (*N* = 3)		Independent *t*-test	
Scales^a^	*M*	SD	*M*	SD	*M*	SD	*P*-value	Hedges’ *g*
Acceptability of intervention measure (AIM) score^b^	3.65	0.57	3.60	0.53	4.53	0.46	.389	1.76
Intervention appropriateness measure (IAM) score	3.78	0.47	3.75	0.46	4.17	0.63	.645	0.89
Feasibility of intervention measure (FIM) score	3.63	0.50	3.63	0.51	3.67	0.29	.113	0.08

^a^Scale range 1–5 (1 = completely disagree; 5 = completely agree); four items per subscale.

^b^Mean score calculated for each subscale.

The coordinating teachers used the toolkit on a ‘needs basis’ and combined their own experience with the ideas from the toolkit. The dose delivered within class was 42% (8/19) across the three schools. School one delivered 32%, school two delivered 21% and school three delivered 26% of the toolkit (Supplementary File 7). Aspects delivered included the problem-solving scenario cards, the public speaking challenge cards, the ASF 30 s icebreaker game, and the communication content planning template.

Between the schools, there were seven monthly check-ins with the intervention group coordinating teachers between December and April 2024. Two of the three schools participated in the first check-in interview during December 2024. School two requested to postpone the first interview as there had not been an opportunity to use the toolkit within the first month due to competing priorities within the Transition Year (i.e. an annual musical and work experience). Similarly, for the January check-in, school two had little opportunity to use the toolkit due to school closures due to national weather warnings and the coordinating teacher being out on parental leave.

After the first monthly check-in, it became clear that an interview script to understand the implementation of the toolkit was premature, as the coordinating teachers did not have sufficient opportunity to engage with the toolkit. Thereafter, the check-ins were reduced to bi-monthly, and they mainly operated as an implementation support opportunity for the coordinating teachers focusing on progress to date with the toolkit, and working together to identify opportunities to use the toolkit going forward.

The lack of implementation can be explained by the time constraints due to the busy Transition Year programme, ‘it was just hard to be consistent with it [the toolkit] because with the Transition Year programme they're missing from school so much’ (Coordinating teacher A, female, School 1). Other factors that influenced the lack of time included the timing of the intervention, the number of public holidays throughout the year, only having one timetabled class within the week, and coordinating teacher absenteeism due to ‘being out on parental leave’ (Coordinating teacher B, male, School 2). Some peer leaders felt that the time constraints led to a lack of organization or rushed efforts.

And we didn't really do it as precisely as we should have…I don't feel like it was thought through as much as it should have…I just feel like we weren't really as organised as we should have been. (Student 1b, female, School 2)

Competing priorities also influenced the delivery of the toolkit, such as the other components within the ASF programme or other subjects where teachers would ‘dedicate more of the time to that whereas active school class it might be one period or two periods a week’ (Coordinating teacher C, male, School 3).

In relation to dose received, there was some confusion as peer leaders found it hard to recall which aspects of the toolkit were used. When asked to describe their efforts to communicate PA messages, peer leaders frequently described the traditional methods within the ASF programme and not that of the COMMUNICATE toolkit.

I have definitely seen that before [30-seconds game]. I'm not sure if we played it or not. (Student 2a, male, School 1)

During the focus groups with the peer leaders, they identified aspects of the toolkit that would have been useful to support their role in implementing the programme.

I think the table one over there [Communication content planning template] is really good because I feel like in class when we were posting everything, we kind of lost track. We would be off, maybe we'd have a bank holiday, then come back, and we didn’t really know where we left off. (Student 1b, female, School 1)

To further justify the need for the toolkit, the peer leaders described the lack of awareness ‘about what Active School Flag actually is’ among junior years and the peer leaders themselves at the start of the year. Additionally, peer leaders described a lack of consistent communication efforts. Rather, one-off methods of sharing messages were adopted, and social media was largely underutilized.

The toolkit was often adapted by the coordinator within each specific school context rather than precisely following the steps from the coordinating teacher’s manual.

I'm trying to fit it into our dynamic in school and what events we were doing or what activities we were running. (Coordinating teacher A, female, School 1)

### Suggested changes to the intervention and study procedures

The coordinating teachers felt there was a lot of information provided in the beginning. Breaking the training up into ‘a specific theme to a zoom’ meeting, using both in-person and online approaches, ensuring material can be accessed in their own time, and using a workshop-style approach to train the coordinating teachers together could provide a more efficient solution going forward as opposed to ‘a big broad toolkit’ (Coordinating teacher C, male, School 3). For example, providing the coordinating teachers with an opportunity to practise using the toolkit with each other and ‘put them in the shoes of a student and have teachers experience what the toolkit is like from a student’s perspective’ (Coordinating teacher A, female, School 1). Another option is to have information that can be accessed within their own time, for example, ‘two or three-minute videos of a teacher kind of doing it with their class and seeing how it actually works in real-life visuals’ (Coordinating teacher A, female, School 1).

To combat the implementation challenges mentioned under objective three, participants suggested some changes to the toolkit to improve overall clarity and quality. During the interviews and focus groups, it became clear that there was some confusion about the purpose of some aspects of the toolkit, such as the purpose of the content planning template and the PA message bank posters. Furthermore, the peer leaders were confused with other similar Transition Year activities and some changes were suggested to improve the clarity and quality of the toolkit. For example, the PA message bank posters were intended to be a fact sheet for the peer leaders to refer to or draw upon, but they interpreted them as posters to stick up on display for the whole school to see.

...reading that [information on the poster] and looking at it in my own hands, it seems like a really good idea, but I feel like if it was on the wall, it would just be a poster that I would walk past. (Student 4b, female, School 2)

Although the toolkit was developed for the ASF programme, further tailoring of the toolkit to ASF may be necessary. For example, one coordinating teacher felt that the toolkit may be more relevant for schools newer to the ASF programme because they ‘already knew all of that [first and second section of the toolkit]’ so they just looked for a ‘new bit…from the toolkit this year’ (Coordinating teacher C, male, School 3), while another felt that different sections within the toolkit may be more relevant to schools at different stages within the ASF programme.

Peer leaders suggested assigning the toolkit to one particular team of peer leaders in the class to ‘put all of our focus into this. And then the other teams could focus on other things’ which might have worked better to ensure it gets done (Student 3a, male, School 1). Timing was discussed as the toolkit and initial introduction session were conducted early November. Feedback from the peer leaders and coordinating teachers indicated that they only looked at the toolkit ‘later on in the year’ which did not leave much time, so going forward it should be introduced from the ‘start of the year’. Table 3 summarizes the changes needed to the intervention and study procedures before proceeding to a definitive trial.

**Table 3:** Summary of the proposed intervention and study procedure changes.

Aspect of the trial	Details of change
Recruitment	Expand the inclusion criteria to all stages of the ASF programme
	Add rotating ASF class as exclusion criteria
Data collection procedures	Move the timing of data collection (beginning of the year and before the last month of the school year, i.e. avoid May)
Outcome measures	Determine validity and reliability of the Determinants of PA Questionnaire, knowledge of PA, and beliefs about the consequences of behaviours, instruments for adolescents
Intervention components	Reduce the number of posters in the toolkit and/or clearly label as ASF class posters or whole-school posters
	Condense instructions in the teacher's manual and/or attach instructions directly to each game or activity
	Map toolkit components to specific aspects of the ASF programme and identify core components and additional components to allow flexibility and adaptations
Intervention training and support	Administer to schools at the start of the year
	Break training up into aspects related to the ASF components
	Assigning toolkit components to the Media and Design team within the ASF class
	Embed implementation support for example, a bi-monthly check-in with the WSP coordinating teacher

### Objective 4: evaluation of resources and ability to manage and implement the study and intervention

Ethical approval was granted from the institutional review board, and the approved procedures and protocols were followed throughout the study. This study was manageable with one full-time PhD student who managed the study and intervention support throughout. Additional support was sought for an assistant moderator to support the focus groups. Training and support required 1 h per school initially and 30 min bi-monthly thereafter for the duration of the intervention for each school. Data collection involved 2 h per school for focus groups; interviews and survey administration took a further 2 h per school, approximately. The study utilized existing university software licence packages, such as the Qualtrics survey platform, SPSS statistical analysis software, and NVivo qualitative analysis software. The printing cost of the toolkit was ∼€13.50 per school. The implementation of the toolkit required the time of the coordinating teachers during the ASF class. The time available for the coordinating teacher to implement the toolkit varied between schools due to competing priorities within the Transition Year and the ASF programme (Supplementary File 7).

### Objective 5: preliminary evaluation of participant responses to intervention

The majority of the sample identified as female (T1: 57.9%, T2: 66.7%) and only 1.6% identified as another gender at baseline. Table 4 presents the between-group and within-group analyses. There were some differences between the treatment groups at baseline. The intervention group had significantly higher teamwork (cooperation) skills [*t*(124) = 3, *g* = 0.06, *P* = .002, 95% confidence interval (CI) (0.13–0.68)], knowledge about PA [*t*(124) = 4.27, *g* = 0.79, *P* < .001, 95% CI (0.42–1.2)], and beliefs about PA [*t*(124) = 2.35, *g* = 0.4, *P* = .01, 95% CI (0.13–0.64)] in comparison to the control group at baseline. There were no significant differences between the groups at follow-up. Within-group analyses revealed significant increases from baseline to follow-up in peer leaders’ communication self-efficacy for both the intervention [*t*(36) = 10.47, *g* = 1.3, *P* < .001, 95% CI (−0.18.77, −12.68)] and control groups [*t*(32) = 9.2, *g* = 1.5, *P* < .001, 95% CI (−19.62, −12.5)]. There were significant increases in peer leaders’ beliefs about the consequences of their behaviours in both the intervention group [*t*(36) = 1.75, *g* = 0.41, *P* = .044, 95% CI (−0.21, 0.48)] and in the control group [*t*(32) = 2.25, *g* = 0.31, *P* = .016, 95% CI (−0.87, −0.04)]. In addition, the control group had significant increases in teamwork advocacy skills [*t*(32) = 2.18, *g* = 0.15, *P* = .019, 95% CI (−0.57, −0.02)] and knowledge about PA [*t*(32) = 1.71, *g* = 0.006, *P* = .049, 95% CI (−0.66, 0.06)].

**Table 4:** Descriptive statistics at baseline and follow-up with independent and paired samples *t*-test results.

Time point	Baseline (T1) (*N* = 126)						Follow-up (T2) (*N* = 99)						Intervention		Control	
Group	Intervention (*n* = 47)		Control (*n* = 79)		T1 independent *t*-test		Intervention (*n* = 49)		Control (*n* = 48)		T2 independent *t*-test		T1–T2 paired *t*-test		T1–T2 paired *t*-test	
	*M*	SD	*M*	SD	*P*-value	*g*	*M*	SD	*M*	SD	*P*-value	*g*	*P*-value	*d*	*P*-value	*d*
Age	15.49	0.83	15.66	0.66	–	–	15.9	0.42	15.96	0.29	–	–	–	–	–	–
Socio-economic status (FAS III)	15.83	2.04	15.39	1.84	–	–	16.08	1.87	15.9	1.81	–	–	–	–	–	–
Communication self-efficacy sum score	42.53	7.56	41.84	9.51	.335	0.078	56.27	12.77	58.46	12.7	.2	0.17	<.001	1.3	<.001	1.5
Teamwork Cooperation skills sum score	4.93	0.71	4.5	0.79	.002	0.06	4.86	0.83	4.79	0.82	.332	0.09	.445	0.09	.437	0.36
Teamwork Advocacy skills sum score	4.04	0.88	3.85	0.87	.114	0.2	3.81	1.05	3.99	0.96	.189	0.18	.195	0.24	.037	0.15
Teamwork Negotiation skills sum score	4.43	0.69	4.23	0.7	.072	0.29	4.5	0.68	4.53	0.73	.395	0.04	.089	0.1	.19	0.39
Health Literacy sum score	32.53	4.13	31.67	3.4	.104	0.2	31.98	4.15	32.88	3.9	.138	0.22	.414	0.13	.184	0.34
Knowledge of PA mean score	5.18	1.18	4.37	0.93	<.001	0.79	5.09	1.21	4.78	1.3	.118	0.25	.214	0.075	.049	0.006
Beliefs about the consequences of behaviour mean score	6.46	0.61	6.08	1.01	.01	0.4	6.12	0.99	6.37	0.76	.087	0.28	.044	0.41	.016	0.31

Participants felt that using the toolkit improved peer leaders’ knowledge about PA and ASF and their personal skill development. The peer leaders did not realize ‘how inactive people actually are’ and how important it is ‘to get everyone active in a way that they'll enjoy’ (Student 4b, female, School 2). In terms of personal skill development, one coordinating teacher was surprised by ‘students who normally wouldn't volunteer themselves…get up and talk in front of classes or in front of people…because they were given the challenge task. And were well able to do it’ (Coordinating teacher A, female, School 1) as a result of the activities in the COMMUNICATE toolkit.

The toolkit was described as a facilitator for the overall running of ASF by coordinating teachers, as it gave them confidence in the peer leaders ‘being able to organise things’ and ‘come up with some solutions’ when a challenge arises (Coordinating teacher C, male, School 3). Furthermore, the toolkit helps to ensure the PA message is communicated clearly with the hope that ‘more will act on that message’ to create a more physically active whole-of-school environment (Coordinating teacher C, male, School 3).

The training and support provided with the COMMUNICATE toolkit for the coordinating teachers was ‘hard kind of put it into place, what it would look like in practice’ but once they started to use the toolkit, ‘it became more clear…what we needed to do and how to use each, each tool, each aspect of it’ (Coordinating teacher A, female, School 1). The frequent check-ins provided guidance and helped clarify the different aspects of the toolkit.

## Discussion

This feasibility of the co-created COMMUNICATE toolkit with the ASF programme implementers was examined. Schools and suitable participants can be recruited. Data collection and the intervention and study procedures were feasible, acceptable, and suitable for participants. The research team managed the evaluation. More time for schools to complete the intervention is required and, finally, the barriers to implementation of the intervention need to be addressed before evidence of impact can be determined. A definitive trial would be feasible after changes are made to the intervention and study procedures.

Recruitment capability compares to similar school-based feasibility studies. The 40% recruitment rate (6/15 schools) is consistent with other studies reporting one to seven schools (Jago *et al.* 2012, Corder *et al.* 2016, Owen *et al.* 2018, Sebire *et al.* 2018, Watkins *et al.* 2024). A systematic review of cluster-randomized feasibility trials found a medium number of eight schools recruited across 24 studies (Parker *et al.* 2022). This study recruited the participants who were available from the sampling pool; however, no single-gender boys’ schools were available to recruit. In Ireland, the majority of secondary schools are mixed gender (70%) whereas single-gender girls and boys account for 15% and 12% respectively (Department of Education and Skills 2025). Future recruitment strategies should consider this distribution spread.

Reaching student recruitment targets in schools is often difficult with 46% of cluster-randomized feasibility trials achieving their goals (Parker *et al.* 2022). Common barriers include lack of time, incentives or alignment with school ethos (Parker *et al.* 2022). The retention rate of peer leaders was high (i.e. 82%) which is comparable to other school-based feasibility studies like PLAN-A (92.5%) and Go-Active (87.3%) (Corder *et al.* 2016, Sebire *et al.* 2018).

Data collection procedures and outcome measures require some improvements to reduce participant burden and ensure accuracy of the data. Surveys were acceptable to both peer leaders and coordinating teachers. Retention rates could be improved by avoiding data collection at the start and end of the year (Hatch *et al.* 2023). Furthermore, due to the nature of the Transition Year students are often absent from the classroom for work placements or volunteering in the community (Smyth *et al.* 2019). It is important to consider the number of student surveys that were incomplete and had to be removed due to missing data. Survey length should be re-examined, as surveys exceeding 20 min are not recommended for this age group (Omrani *et al.* 2019).

The Determinants of PA Questionnaire knowledge and beliefs about PA were problematic. A recent scoping review synthesized and critiqued the available instruments and possible outcome measures to evaluate the communication of PA messages (Grady *et al.* 2025a). There were many instruments measuring the same outcome measure and a lack of psychometric properties for these instruments, thus, were not appropriate to assess the outcome of interest for this study. The Determinants of PA Questionnaire has not been validated for use among this population, but it was determined to be most suitable for the purpose of this study. Upon examination, the one positively worded item in this scale appeared most problematic; nevertheless, after removing the item, the scale still did not have good internal consistency. Omrani *et al.* (2019) highlighted that adolescents are less able to respond to negatively worded items; thus, the mix of both negative and positively worded items was problematic. In the future, a process of adapting and establishing validity and reliability of the instrument is needed.

Low implementation was primarily due to time constraints, a common implementation barrier (Naylor *et al.* 2015, McHale *et al.* 2022). Bi-monthly check-ins were vital for monitoring dose, one of the most commonly assessed feasibility objectives, and providing support (Chan *et al.* 2017). In contrast, a similar feasibility study that was delivered over one school term (i.e. 10 weeks) showed that almost all intervention components were delivered as intended. However, peer leaders were trained by the research team (Nathan *et al.* 2017). The discrepancy between the COMMUNICATE toolkit’s planned and delivered intervention components is noteworthy. Consideration for the level of support required for schools and teachers to effectively engage with the delivery of complex, evidence-based interventions is required. Ultimately, the low dose delivered in this study confirms that the COMMUNICATE toolkit is a complex ‘real-world’ intervention that may require more time and several delivery attempts before the implementers are confident in delivering the intervention. Through the co-creation of the COMMUNICATE toolkit, consideration for adapting the toolkit within each school context and the practical circumstances of embedding a toolkit within an existing WSP were taken (Lane *et al.* 2021, Jago *et al.* 2023, Grady *et al.* 2025c). However, feedback indicated the need to align the toolkit with the ASF programme even further and to allow flexibility to adapt during delivery. This study further highlights the need to move away from a standardized approach towards offering schools the autonomy and flexibility to create appropriate and successful interventions (Jago *et al.* 2023). To establish the true value of the COMMUNICATE toolkit and the use of each element, it is necessary to observe its use over time as part of a WSP, such as the ASF. Furthermore, more work is needed to develop and refine implementation strategies for embedding an intervention component within an existing WSP.

A small research team managed this study, as the intervention was delivered by the coordinating teachers in each respective school as part of the ASF programme. When implemented as part of a WSP, the COMMUNICATE toolkit is a low-cost intervention that requires minimal resources to implement from both a research and policy perspective, which emphasizes the potential for scalability and sustainable real-world implementation. The researchers trained and provided implementation support to the coordinating teachers to use the toolkit and managed data collection and analysis. This ‘real-world’ implementation considers each school context and future intervention scale-up rather than solely relying on individuals highly skilled in intervention delivery (Lane *et al.* 2021, Jago *et al.* 2023). This study relied on the coordinating teacher in each school to reply to emails and to facilitate data collection, which is largely outside the control of the research team. Future research should consider exploring reducing the potential burden placed on teachers when facilitating research interventions in the school setting.

Preliminary participant responses revealed improvements in both treatment groups regarding communication and teamwork skills, health literacy, PA knowledge, and beliefs. Notably, both the intervention and control groups were required to develop a communications strategy as part of the ASF programme, and the control group received some minimal general guidance (Supplementary File 1). The intervention group appreciated the physical resources and activities to accelerate the development of these skills. It is unclear whether the improvements in both treatment groups can be explained by existing opportunities for the Transition Year students in the schools or the lack of time to implement the COMMUNICATE toolkit. Responses suggest that the toolkit provides the relevant content to develop those skills during ASF class time. The potential benefits of the toolkit for the wider school community such as increased knowledge, awareness, and PA behaviour should be explored in future research (Grady *et al.* 2025b).

### Strengths and limitations

The robust feasibility trial design and use of mixed methods strengthened this study. Using quantitative data alone would not have provided the full context regarding the reasons for low implementation. The bi-monthly check-ins were crucial to understand real-time implementation to avoid retrospective recalling at the end of the intervention. Additionally, this feasibility study includes cost data which improves the overall transparency and reproducibility of the study.

Some limitations should be considered when interpreting the findings. The same researcher delivered the training, provided support and conducted the follow-up interviews and focus groups to understand the acceptability of the toolkit, which may have raised some bias. Separate researchers conducting the research and implementation support should be considered. Readers should interpret the findings in light of the purposive sampling procedures employed in this study. Allocation to the intervention group was based on proximity to the university rather than random allocation. Therefore, the findings should be interpreted as exploratory and preliminary. Furthermore, recruitment for the focus group discussions may have introduced volunteer bias. To assess the suitability of the procedures and outcomes measured, a survey instrument could be included as both peer leaders and coordinating teachers struggled to recall the instrument. Refinement of the eligibility criteria is needed to ensure the exclusion of a rotating class of peer leaders and the opportunity to receive the full intervention dose is provided. Finally, it is important to recognize the proportion of respondents excluded from analyses due to missing or spoilt data and outliers (12% at baseline and 17% at follow-up).

## Conclusions

In summary, the COMMUNICATE toolkit is a valuable, but complex, resource requiring refinement and flexibility for diverse school settings. This feasibility study was a crucial step in finalizing the development of the COMMUNICATE toolkit and ultimately complemented the co-creation process. There are many key learnings from this study for research, practice, and policy. Firstly, the need for the toolkit and WSP in general to be flexible and adaptive in nature to account for each school as its own complex adaptive subsystem. Additionally, the well-cited factor of time as an implementation barrier should be considered in relation to its implications for WSPs and the delivery of the COMMUNICATE toolkit. Both peer leaders and coordinating teachers recognized, the value the COMMUNICATE toolkit provides to support the WSP. The findings suggest that the COMMUNICATE toolkit and the study procedures should be refined based on the feedback from this study and tested as part of an internal pilot before proceeding to the definitive trial.

## Supplementary Material

daag110_Supplementary_Data

## Data Availability

The data underlying this article will be shared on reasonable request with the corresponding author.
