# Supplementary material for: The feasibility of the COMMUNICATE toolkit to support the communication of physical activity messages with adolescents in Irish schools
Source: Health Promot Int. 2026 Aug 6;41(4):daag110. doi: 10.1093/heapro/daag110 (PMC13445620; doi:10.1093/heapro/daag110)
Supplement: Supplementary file 1 [file daag110_supplementary_data.docx]

**Supplementary materials for ‘The Feasibility of the COMMUNICATE toolkit to support the communication of physical activity messages with adolescents in Irish schools’**

**Supplementary file 1- The Active School Flag Did You Know? Communications Campaign guidelines**

Did You Know? Campaigns are information campaigns that may be run on a weekly, fortnightly or monthly basis or at a specific time during the year.

Purpose of the campaigns:

1. To share messages from the ASF class
2. To share information about upcoming physical activity events
3. To share general information about physical activity

Suggested Did You Know? Messages:

The following are suggestions of important Did You Know? messages:

- We’re working towards the Active Flag
- Physical Activity Guidelines: 60-minutes per day
- 10, 000 steps per day, tracking steps
- Ways to be active in our school
- We have a school walkway
- Ways to be active in our local community
- Mapped walking/running routes in the local community
- Benefits of physical activity for learning
- Benefits of physical activity for wellbeing

Important considerations:

- Decide on the best channel (e.g., school newsletter, posters, video, social media) for promoting your messages or possible create a multi-channel campaign
- Review the effects of each campaign. This may require some consultation with groups of students to gauge their awareness of the campaign and their understanding of its key messages.

Steps to an informative campaign:

While some of the campaigns will be unique and specific to each school and its local community, some will involve sharing messages about general physical activity. The following steps will help to create the most informative Did You Know? Campaigns.

- Research the benefits of physical activity for teenagers paying particular attention to benefits which are most appealing to teenagers
- Examine national and international campaigns and studies which highlight the benefits of physical activity for teenagers e.g. Her Moves
- Review the findings of the whole-school questionnaire in relation to PA outside of school (e.g., local sports clubs, recreational activities, etc.)
- Consult with the local sports partnership to find out what physical activity opportunities they are supporting and promoting for teenagers locally
- Consider and promote any additional physical activity opportunities which may be taking place in the community (e.g., park run, local charity cycle, darkness into light).

**Supplementary file 2- CONSORT 2010 checklist of information to include when reporting a pilot or feasibility trial**

| **Section/ Topic** | **Item No** | **Checklist item** | **Reported on page No** |
| --- | --- | --- | --- |
| **Title and abstract** | | | |
|  | 1a | Identification as a pilot or feasibility randomised trial in the title | 1 |
|  | 1b | Structured summary of pilot trial design, methods, results, and conclusions | 1-2 |
| **Introduction** | | | |
| Background and objectives | 2a | Scientific background and explanation of rationale for future definitive trial, and reasons for randomised pilot trial | 2-4 |
|  | 2b | Specific objectives or research questions for pilot trial | 4 |
| **Methods** | | | |
| Trial design | 3a | Description of pilot trial design (such as parallel, factorial) including allocation ratio | 4 |
|  | 3b | Important changes to methods after pilot trial commencement (such as eligibility criteria), with reasons | N/A |
| Participants | 4a | Eligibility criteria for participants | 4 |
|  | 4b | Settings and locations where the data were collected | 5-6 |
|  | 4c | How participants were identified and consented | 4-5 |
| Interventions | 5 | The interventions for each group with sufficient details to allow replication, including how and when they were actually administered | 4-5, Supplementary file 2 |
| Outcomes | 6a | Completely defined prespecified assessments or measurements to address each pilot trial objective specified in 2b, including how and when they were assessed | 6-7, Supplementary file 6 |
|  | 6b | Any changes to pilot trial assessments or measurements after the pilot trial commenced, with reasons | N/A |
|  | 6c | If applicable, prespecified criteria used to judge whether, or how, to proceed with future definitive trial | Supplementary file 6 |
| Sample size | 7a | Rationale for numbers in the pilot trial | 4 |
|  | 7b | When applicable, explanation of any interim analyses and stopping guidelines | N/A |
| Randomisation: |  |  |  |
| Sequence  generation | 8a | Method used to generate the random allocation sequence | N/A |
|  | 8b | Type of randomisation(s); details of any restriction (such as blocking and block size) | N/A |
| Allocation  concealment  mechanism | 9 | Mechanism used to implement the random allocation sequence (such as sequentially numbered containers), describing any steps taken to conceal the sequence until interventions were assigned | N/A |
| Implementation | 10 | Who generated the random allocation sequence, who enrolled participants, and who assigned participants to interventions | N/A |
| Blinding | 11a | If done, who was blinded after assignment to interventions (for example, participants, care providers, those assessing outcomes) and how | N/A |
|  | 11b | If relevant, description of the similarity of interventions | N/A |
| Statistical methods | 12 | Methods used to address each pilot trial objective whether qualitative or quantitative | N/A |
| **Results** | | | |
| Participant flow (a diagram is strongly recommended) | 13a | For each group, the numbers of participants who were approached and/or assessed for eligibility, randomly assigned, received intended treatment, and were assessed for each objective | 4-5, 8 |
|  | 13b | For each group, losses and exclusions after randomisation, together with reasons | 4-5, 8 |
| Recruitment | 14a | Dates defining the periods of recruitment and follow-up | 5-6 |
|  | 14b | Why the pilot trial ended or was stopped | 5-6 |
| Baseline data | 15 | A table showing baseline demographic and clinical characteristics for each group | 13-14 |
| Numbers analysed | 16 | For each objective, number of participants (denominator) included in each analysis. If relevant, these numbers should be by randomised group | 4, 8, 13-14 |
| Outcomes and estimation | 17 | For each objective, results including expressions of uncertainty (such as 95% confidence interval) for any estimates. If relevant, these results should be by randomised group | 8-16 |
| Ancillary analyses | 18 | Results of any other analyses performed that could be used to inform the future definitive trial | N/A |
| Harms | 19 | All important harms or unintended effects in each group | 13-16 |
|  | 19a | If relevant, other important unintended consequences | N/A |
| **Discussion** | | | |
| Limitations | 20 | Pilot trial limitations, addressing sources of potential bias and remaining uncertainty about feasibility | 16 |
| Generalisability | 21 | Generalisability (applicability) of pilot trial methods and findings to future definitive trial and other studies | 16 |
| Interpretation | 22 | Interpretation consistent with pilot trial objectives and findings, balancing potential benefits and harms, and considering other relevant evidence | 14-17 |
|  | 22a | Implications for progression from pilot to future definitive trial, including any proposed amendments | 12, 17 |
| **Other information** | | |  |
| Registration | 23 | Registration number for pilot trial and name of trial registry | N/A |
| Protocol | 24 | Where the pilot trial protocol can be accessed, if available | N/A |
| Funding | 25 | Sources of funding and other support (such as supply of drugs), role of funders | 17 |
| Ethical approval | 26 | Ethical approval or approval by research review committee, confirmed with reference number | 4 |

**Supplementary file 3- Fidelity checklist**

School No.: ______________

Check-in No.: ______________

Date: ______________

| **Toolkit components** | **Component delivered (dose)** | **Adherence to manual** |
| --- | --- | --- |
| **Area 1: Putting more emphasis on ASF** | ✔/x | ✔/x |
| Played the introduction video for the ASF class (ASF website) |  |  |
| The ASF 30-seconds game |  |  |
| Raised awareness about ASF (e.g. sharing survey data with the rest of the school) |  |  |
| Created active campaign videos to encourage people to be more active |  |  |
| The coordinator identified and/or organised guest speakers |  |  |
| **Toolkit components** | **Component delivered (dose)** | **Adherence to manual** |
| **Area 2: Planning the promotion of physical activity** | ✔/x | ✔/x |
| Reviewed previous ASF communication efforts |  |  |
| Class brainstorming activities |  |  |
| Filled out the content planning template |  |  |
| Shared tips to boost social media video |  |  |
| Physical activity message development or sharing of existing messages |  |  |
| Covered the general tips for promoting physical activity (do’s and don’ts) |  |  |
| **Toolkit components** | **Component delivered (dose)** | **Adherence to manual** |
| **Area 3: Skill development** | ✔/x | ✔/x |
| Identifying class skills needs |  |  |
| Listening activities |  |  |
| Played tips and tricks for presenting and public speaking video |  |  |
| Challenge cards (public speaking activity) |  |  |
| Discussed problem-solving steps |  |  |
| Scenario cards (problem-solving and decision-making activity) |  |  |
| Identifying stakeholders to work with outside of the ASF class |  |  |
| Common component among all areas |  |  |
| Discussions about being inclusive and diverse |  |  |

**Supplementary file 4- Final semi-structured interview guides**

**Teacher interviews**

**Questions related to the acceptability and suitability of the intervention (objective 3):**

1. What was your experience of receiving the COMMUNICATE toolkit as the ASF coordinator?
a. Can you share your thoughts on the support and training provided for the COMMUNICATE toolkit
b. In your opinion, how does the toolkit fit within the ASF class?
c. You filled out a survey before this interview asking you some questions about your satisfaction with, the fit and success of the toolkit within your school. The other coordinators piloting the toolkit also filled this out. I had a quick look at the responses and calculated some averages. The agreeable, palatable and satisfactory nature of the COMMUNICATE toolkit in ASF schools scored an average X out of 5. In your opinion, how could we make this better?
2. Can you tell me about how and when you used the COMMUNICATE toolkit?
a. What opportunities presented themselves?
b. Look at the checklist at the back of the toolkit, what is your opinion on each of the different aspects? Like/dislike? Why?
c. How satisfied were you with the toolkit? with the process undertaken to implement the toolkit in your school?
d. In the survey you filled out earlier the perceived fit, relevance and compatibility of the toolkit for improving the communication of physical activity in ASF schools scored an average of X out of 5. How could we improve this?
3. Schools are very busy places and TY in particular is very busy. Given everything in your workload, what motivated you to use the COMMUNICATE toolkit?
a. What was your capacity to use the toolkit? i.e. time
b. What factors influenced your decision?
c. The final result from the survey I would like to share with you was about the extent to which the COMMUNICATE toolkit could be successfully carried out in ASF schools which scored an average of X out of 5. How could this be improved?

Questions related to data collection procedures and outcome measures (objective 2):

4. I want to ask about your experience with the research component, this included surveys, focus groups/interviews, monthly check-ins from October to now. Can you describe to me what was your experience with the research from November (when we first met) to now?
a. How did you feel about the amount of effort required to complete surveys, monthly check-ins, interviews, etc.
b. What was the level of clarity for i) you and ii) the students with the surveys, monthly check-ins, interviews, etc?

Questions related to preliminary evaluation (objective 5):

5. In your opinion, what is the impact of the COMMUNICATE toolkit for ASF schools?
a. For you as the ASF coordinator- how does the COMMUNICATE toolkit influence your role?
b. For the ASF TY students- how does the COMMUNICATE toolkit influence their roles?
c. For the whole-school community- how does the COMMUNICATE toolkit influence their day-to-day lives?

E.g. confidence, communication skills, knowledge and awareness, physical activity levels?

6. One final million-dollar question, if you could make one request to improve the toolkit for other ASF schools what would that be?
7. Final question about your ASF progress this year and plans for next year?

**Student focus group script:**

1. One of the activities you are expected to complete in the Active School Flag class is to run a ‘Did You Know??’ or communications campaign. Can you tell me about your experience with that this year.

Prompts:

- What messages did you communicate? How did you decide this?
- Where did you do your communications? i.e. platforms, intercom, class visits
- When did you do your communications? i.e. time of day, particular day of the week, specific time of the year
- Who did the communicating? How did you decide this?
- How did you make sure they understood/got the message? i.e. strategies, mechanisms, evaluations
- What do you think went well? Why?
- What do you think did not go well? Why?

This is a box of resources that I gave to your teacher at the beginning of the year to help the ASF class communicate physical activity messages. I am going to pull out some of the resources and we will pass them around the room until everyone has had an opportunity to see them all.

2. Before passing them around, can you please tell me about when you have seen this toolkit before?

[Give each person one resource] Take a minute to look at this resource and think about when you may have seen it before. When you are finished looking, pass yours to the right [Continue until all resources have been reviewed].

Now can anyone tell me about your experience with these resources? (Objective 3: Acceptability and suitability of intervention)

Prompts:

- If used: when and how?  What was your opinion of using it? What helped you when you used these resources? What might have prevented you from using these resources?
- If not used: do you have any questions about them?  What might have prevented you from using these resources? (if necessary, explain its purpose), Is there anything in this box that would have helped you with your efforts to communicate physical activity? how?

3. How do you feel about the overall idea of using the toolkit within the ASF class to help you communicate with the rest of the school? (Objective 3: acceptability & suitability of intervention)

Prompts:

- Does it fit within the ASF class? Practicality, time?
- Positive response- what aspects did you like? why? Is there anything that would make it better?
- Negative response- what aspects did you dislike? why? How could it be improved?
- Is there anything you would change?
- Is there anything missing? (refer to Q1 prompt answers to what went well/didn’t this year- Could the toolkit provide something to help with that)

4. In your opinion, how successful/unsuccessful is the COMMUNICATE toolkit for i) you, ii) the ASF class, iii) the rest of the school? (Objective 5: preliminary evaluation)

Prompts:

- What impact does it have for you as a peer leader in the ASF class?
- What impact does it have for the ASF coordinators?
- What impact does it have for the rest of the school community?
- E.g. confidence to communicate, knowledge and awareness levels, PA levels, engagement with ASF

5. As part of the research process, you filled out a survey in November and again this week and you took part in this focus group. Describe your experience with this research process from November to now. (Objective 2: data collection procedures and outcome measures)

Prompts:

- Consent
- Time/ burden
- Clarity of the questions

**Supplementary file 5- Predefined Orsmond and Cohn (2015) data collection points**

| **Objective 1: Evaluation of recruitment capability and resulting sample characteristics** | |
| --- | --- |
| **Main question: Can we recruit appropriate participants?** | **Data sources** |
| 1. How many potential eligible members of the targeted population are accessible in the local community? | Not applicable as recruitment was at the school level. The number of available schools were recorded. |
| 2. What are the recruitment rates? a. How many participants enter the study at a time b. How long does it take to recruit enough participants into the study? c. What are the refusal rates for participation? | a. Not applicable, all participants have to start at the same time due to the induction session. Number of schools contacted, number of schools that responded, their response, whether they met eligibility criteria b. Record start and end date of recruitment c. Number of refusals and rationale were recorded in recruitment record. |
| 3. How feasible and suitable are eligibility criteria? a. Are criteria clear and sufficient or too inclusive and restrictive? | Recruitment records will note number of coordinators who expressed interest, met eligibility criteria, and consented to participate. The research team will review the eligibility criteria at the end of the intervention |
| 4. What are the obstacles to recruitment? a. Are colleagues and local organisations willing to assist with recruitment? b. What are the reasons for refusal or ineligibility? | a. Interviews with coordinators about data collection b. Any reason given for refusal or ineligibility were recorded |
| 5. How relevant is the intervention to the intended population? a. Do study participants show evidence of need for the intervention? b. Are the characteristics of the study participants consistent with the range of expected characteristics as informed by the research literature? | a. Baseline self-reported skills and competencies from survey and data from interviews and focus groups b. Participants' characteristics were examined within the survey, including age, gender, school type, and socio-economic status.  During the focus groups and interviews, participants’ responses to study recruitment were captured and explored further. |
| **Objective 2: Evaluation and refinement of data collection procedures and outcome measures** | |
| **Main Question: How appropriate are the data collection procedures and outcome measures for the intended population and purpose of the study?** | **Data sources** |
| 1. How feasible and suitable are the data collection procedures?   a. Do participants understand the questions and other data collection procedures? b. Do they respond with missing or unusable data? | a. Participants were asked to describe their experience with the research process i.e. surveys, monthly check-ins, and interviews. b. Number of missing and unusable data will be calculated |
| 2. How feasible and suitable is the amount of data collection?   a. Do the participants have the capacity to complete the data collection procedures? b. Does the overall data collection plan involve a reasonable amount of time or does it create a burden for the participants? | a. Number and proportion of participants who complete all data collection measures. Follow up in focus groups and interviews b. Total time of each assessment recorded. Asked in focus groups and interviews. |
| 3. Do the measures appear to be performing in a consistent way with the intended population as compared to measurement information available in the research literature? a. Are internal consistency indicators of measures with the recruited sample congruent with expectations based on prior studies reported in the research literature? b. Do planned outcome measures appear to be sensitive to the effects of the intervention? c. Does a suitable outcome measure need to be developed? | The performance of the outcome measures was assessed post hoc, examining the internal validity of and magnitude of effect for each scale. |
| **Objective 3: Evaluation of acceptability and suitability of intervention and study procedures** | |
| **Main Question: Are study procedures and intervention suitable for and acceptable to participants?** | **Data sources** |
| 1. What are the retention and follow-up rates as the participants move through the study and intervention? | Recruitment records will note the number of participants who started and the number who finished the study. |
| 2. What are the adherence rates to study procedures, intervention attendance, and engagement?   a. Does the intervention fit with the daily life activities of study participants? b. Do the participants have enough time and capacity to complete the intervention? c. Does the intervention involve a reasonable amount of time or does it create a burden for the participants? d. To what extent is the intervention acceptable and appealing to participants? e. If appropriate, how many participants agree to be randomised to group? | a. The surveys, researcher records, interviews, and focus groups were used to understand the fit, time, and capacity requirements. b. A fidelity checklist was used during the monthly check-in interviews to establish what aspects of the intervention were implemented and whether they were implemented as intended (Supplementary file 4). Followed up in focus groups and interviews c. Retention rates were reported based on the number of participants who completed both baseline and follow-up measures and the dropout rates. Adherence rates were assessed through the monthly check-in interviews with the coordinator at each of the intervention schools, during which the specific use of the toolkit was discussed to determine how it was delivered (fidelity). Focus groups and interviews were used to ask participants about their perceptions of the intervention. d. The Weiner et al. (2017) implementation questionnaire was used for the teachers and students to determine the acceptability, appropriateness, and feasibility of the COMMUNICATE toolkit post intervention. Focus groups and interviews were used to further probe responses about the acceptability of the intervention. e. N/A |
| 3. What is the level of safety of the procedures in the intervention?  a. Are there any unexpected adverse events? | Researcher records will note any unexpected or adverse events when in contact with study participants. However, as the intervention is delivered during normal class time by the class teacher there is little expected harm outside of normal daily school life. During the focus groups when the researcher is in direct contact with the participants, normal school policy and safeguarding procedures will be followed. Any unintended consequences of the intervention to participants' daily lives will be explored in the interviews and focus groups. |
| **Objective 4: Evaluation of resources and ability to manage and implement the study and intervention** | |
| **Main Question: Does the research team have the resources and ability to manage the study and intervention?** | **Data sources** |
| 1. Does the research team have the administrative capacity, expertise, skills, space, and time to conduct the study and intervention? | The researcher logged any challenges faced with ethical procedures, budgetary allowance, technology and equipment, and data entry and analysis. |
| 2. Can we conduct the study procedures and intervention in an ethical manner?   a. To what extent does staff comply with the approved human participants’ protocol? b. How effectively are adverse events during implementation identified, documented, and reported? | a. Protocols outlined in the ethics application will be reviewed and any evidence on compliance were noted b. Research team members reviewed the process at the end of the intervention |
| 3. Can the study and intervention be conducted within the designated budget? | Record of expenditure was compared to funding awarded. |
| 4. Is the technology and equipment sufficient to conduct the study and intervention, including collection, management, and analysis of data?   a. Is equipment available when needed? b. What is involved in training personal and/or participants to use the equipment? | a. Research team kept a log of any challenges encountered throughout the study. b. Research team received training in qualitative training through modules and workshops. No training required for surveys. |
| 5. Are we able to efficiently and effectively manage data entry and analysis? | Research team kept a log of any challenges encountered. |
| **Objective 5: Preliminary evaluation of participant responses to intervention** | |
| **Main Question: Does the intervention show promise of being successful with the intended population?** | **Data sources** |
| 2. Does examination of quantitative data suggest that the intervention is likely to be successful?  a. Does examination of the data at the participant level suggest that changes in key outcome variables occurred? b. Are the changes of the outcome variable(s) in the expected direction? c. Do the estimates of effects suggest that the intervention has promise? | Research team reviewed results and drew conclusions accordingly.   a. Key outcomes measures (i.e., peer leaders communication self-efficacy, teamwork skills, health literacy, knowledge of PA and beliefs about the consequences of behaviour) were assessed by comparing means pre vs post, intervention vs control. Follow-up interviews or focus groups with the sub-sample of participants from the intervention group were used to evaluate participants’ responses to the intervention. b. Improvements in key outcome measures are expected for both groups however, a larger effect size is expected in the intervention group for the primary outcome measure (Cohen, 1988) c. The intervention effects will be examined after the intervention |
| 2. Do participants or relevant others provide qualitative feedback that may be indicative of the likelihood that the intervention will be successful? | Focus groups and interviews were conducted with students and coordinators. The findings were reviewed by the research team. |
| 4. If the quantitative and/or qualitative data suggest that the intervention is not promising: a. Are the data collection procedures and outcome measures appropriate for the population and study? b. Are the outcome measures and intervention theoretically aligned? c. Is there evidence that the intervention does not produce change in the desired outcomes? d. Is there evidence that the intervention was not implemented in the intended manner? e. Have too many adaptations been made in the intervention process to adequately assess the participants’ responses to the intervention? f. Are the findings congruent with the proposed theoretical model for the intervention? | Results were reviewed by the research team. |

**Supplementary file 6. Internal consistency and magnitude of outcome measures among the total sample**

|  | **Internal consistency** | | **Paired samples t-test** | | | | | |
| --- | --- | --- | --- | --- | --- | --- | --- | --- |
|  | **T1 (N=126)** | **T2 (N=98)** | **T1 (N=70)** | | **T2 (N=70)** | | **T1-T2 (n= 70)** | |
| **Scale** | **α** | **α** | **M** | **SD** | **M** | **SD** | **D** | ***p*** |
| **Communication self-efficacy** | .877 | .909 | 42.04 | 7.91 | 57.93 | 10.95 | 1.66 | <.001 |
| **Teamwork Cooperation** | .894 | .932 | 4.81 | .74 | 4.86 | 0.83 | .06 | <.001 |
| **Teamwork Advocacy skills** | .872 | .916 | 3.89 | .89 | 3.97 | 1.03 | .08 | <.001 |
| **Teamwork Negotiation skills** | .719 | .794 | 4.34 | .69 | 4.49 | .7 | .22 | <.001 |
| **Health Literacy** | .797 | .842 | 32.19 | 3.39 | 32.67 | 3.89 | .13 | <.001 |
| **Knowledge of PA** | .426 | .593 | 4.95 | 1.11 | 5.02 | 1.26 | .06 | <.001 |
| **Beliefs about the consequences of behaviour** | .768 | .889 | 6.21 | .99 | 6.27 | .86 | .06 | .025 |

**Supplementary file 7. Summary of check-in interviews with intervention group coordinators**

|  | **Check-in 1** | **Check-in 2** | **Check-in 3** |
| --- | --- | --- | --- |
| **School 1** | | | |
| **Date of interview** | 11/12/2024 | 11/03/2025 | X |
| **No. of opportunities to use the toolkit (i.e., classes)** | 1 | 5 | 0 |
| **No. of elements of the toolkit used (N=19)** | 1/ Intro to ASF class video  2/ Raised awareness about ASF  3/ Created active campaign videos/ ASF 30- seconds game | 1/ Reviewed previous ASF communication efforts  2/ Challenge cards  3/ Scenario cards | 0 |
| **Total dose delivered** | 32% | | |
| **School 2** | | | |
| **Date of interview** | X | 13/02/2025 | 09/04/2025 |
| **No. of opportunities to use the toolkit (i.e., classes)** | 0 | 2 | 2 |
| **No. of elements of the toolkit used (N=19)** | 0 | 1/ Intro to ASF class video  2/ Raised awareness about ASF  3/ Created active campaign videos | 1/ Physical activity message sharing |
| **Total dose delivered** | 21% | | |
| **School 3** | | | |
| **Date of interview** | 11/12/2024 | 13/02/2025 | 10/04/2025 |
| **No. of opportunities to use the toolkit (i.e., classes)** | 1 | 3 | ~6 |
| **No. of elements of the toolkit used (N=19)** | 1/ Intro to ASF class video  2/ Raised awareness about ASF | 1/ PA message banks | 1/ Challenge cards  2/ Scenario cards |
| **Total dose delivered** | 26% | | |
